# Reduced RING finger protein 10 expression in macrophages is associated with aging‐related inflammation

**DOI:** 10.1002/2211-5463.13049

**Published:** 2021-01-13

**Authors:** Xinyuan Cao, Lidan Liu, Yueyi Zhang, Yingyun Yang

**Affiliations:** ^1^ Department of Gastroenterology Peking Union Medical College Hospital (PUMCH) Chinese Academy of Medical Sciences & Peking Union Medical College (CAMS & PUMC) Beijing China; ^2^ The Southern Medical District of Chinese PLA General Hospital Beijing China

**Keywords:** E3 ubiquitin ligase, immunosenescence, inflammation, macrophages, RNF10

## Abstract

Age‐associated decline of the immune system is referred to as immunosenescence. The E3 ligase RING finger 10 (RNF10) has long been associated with the innate immune response, although a potential role in immunosenescence has not previously been reported. In the present study, we identified that RNF10 expression is lower in aged mouse macrophages than in young cells. After lipopolysaccharide stimulation, RNF10 expression remained at a basal low level in aged mouse cells, but declined sharply in young mouse cells. Knockdown of RNF10 enhanced both the nuclear factor‐κB and interferon regulatory factor 3 signaling pathways and thus enhanced proinflammatory cytokines and type I interferons in macrophages, promoting clearance of *Listeria monocytogenes*. These findings indicate that dysregulated expression of RNF10 is associated with age‐associated immune dysfunction, and RNF10 may thus be a potential target for the treatment of age‐related inflammatory diseases.

## Graphical abstract

The understanding of the mechanism governing the onset of immunosenescence remains incomplete. Here, we found that RNF10 could be suppressed by increased TLR‐TRIF pathway activation and maintained at a low level in aged macrophages compared with young cells. Moreover, reduced RNF10 increased lipopolysaccharide‐triggered signaling pathways of nuclear factor‐κB and interferon regulatory factor 3, thereby enhancing proinflammatory cytokines and interferons in aged cells.

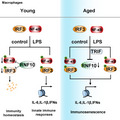

**Abbreviations**
IFN: interferon
IL: interleukin
IRF3: interferon regulatory factor 3
LPS: lipopolysaccharide
NF‐κB: nuclear factor‐κB
RNF10: RING finger 10
siRNA: small interfering RNA
TLR: Toll‐like receptor
TNF: tumor necrosis factor
TRIF: Toll/IL‐1 receptor domain‐containing adaptor inducing IFN‐β

The immune system and the aging process comprise a most intricate and complicated biological phenomena, interacting in a comprehensive approach termed ‘immunosenescence’. Immunosenescence not only occupies a vital position at the frontiers of science, but also has a strong application in clinical practice. Claudio Franceschi first advanced the concept that the aging process was associated with chronic inflammation with an increased level of proinflammatory cytokines [1, 2, 3]. Although the elderly usually demonstrate a low level of inflammation, they are prone to infectious diseases because of an age‐related weakening of immunity. Such an interesting phenomenon has fueled research into immunosenescence. One markable characteristic of immunosenescence is the shifting pattern of immune cells and proinflammatory cytokines. Many approaches have implied that T cells, B cells, dendritic cells (DC), neutrophils and macrophages exhibit differences between old and young individuals [4, 5]. Also, proinflammatory cytokines such as tumor necrosis factor‐α (TNF‐α), interleukin (IL)‐6), IL‐1 and interferon (IFN)‐γ were reported to be involved in aging diseases [6]. The above findings provided mechanistic insights into age‐related clinical implications from the aspects of infectious diseases [7], degenerative diseases [8, 9] and vaccination [7]. Consequently, the discovery of new mediators in immunosenescence should offer new knowledge for drug targets, as well as clinical therapies.

As one of the most prevailing post‐translational modifications, ubiquitination involves the conjugation of ubiquitin to various proteins at lysine residues [10]. Additionally, ubiquitin on substrates varies in poly‐linkages and functions through Ub‐activating enzymes (E1s), Ub‐conjugating enzymes (E2s) and Ub ligases (E3s) [11]. Among the E3 ligases, RNF family members display multiple roles in various aspects of cellular processes, such as DNA repair [12], signal transduction [13], inflammatory and immune diseases [14], apoptosis [15], and metabolism status [11]. Hence, ubiquitin regulation is an indispensable part of individual development and the immune system.

Ring finger protein 10 (RNF10), a member of the RING finger family belonging to E3 ubiquitin ligase, was first discovered in 2000 [16]. With the function of recruiting E2 ligases and target proteins, it was reported to be involved in hyperproliferation, apoptosis, cell differentiation, neuronal development and other biological processes [17, 18, 19, 20]. A previous study showed that overexpression of RNF10 could suppress inflammation by reducing the levels of nuclear factor‐κB (NF‐κB) protein [21], although little is known about its role in immunosenescence. Here, we found that dysregulated expression of RNF10 in macrophages of aged mice is involved in regulating lipopolysaccharide (LPS)‐induced proinflammatory factors, indicating that RNF10 may be a potential target for the therapy of age‐related inflammatory diseases.

## Materials and methods

### Mice and cells

Young (2‐month‐old) and aged (20‐month‐old) male C57BL/6J mice were obtained from the Institute of Zoological Sciences, Chinese Academy of Medical Sciences (Beijing, China). Animals were housed individually under controlled temperature (20–26 °C), humidity (40–70%) and a 12 : 12 h light/dark photocycle (lights on 07.00 h) under specific pathogen‐free conditions. Mice were provided *ad libitum* access to commercial mouse chow and water. All animal experiments were performed in accordance with the National Institutes of Health Guide for the Care and Use of Laboratory Animals and with approval of the Scientific Investigation Board of Chinese Academy of Medical Sciences. Primary peritoneal macrophages were acquired from the peritoneal lavage fluids of mice that were intraperitoneally injected with 3% thioglycollate 72 h and were maintained in 1640 supplemented with 10% fetal bovine serum. Bone marrow‐derived DCs were generated by the addition of recombinant mouse granulocyte macrophage‐colony‐stimulating factor (10 ng·mL^−1^) (PeproTech, Rocky Hill, NJ, USA) and IL‐4 (1 ng·mL^−1^) (PeproTech) to 1640 medium for 5 days. Splenic natural killer cells, CD4^+^ T cells, CD8^+^ T cells and B cells were purified by magnetic‐activated cell sorting (Miltenyi Biotec, Bergisch Gladbach, Germany); cell purity was > 90%. RAW264.7 cells and HEK293 cells (American Type Culture Collection, Manassas, VA, USA) were cultured in Dulbecco’s modified Eagle’s medium containing 10% fetal bovine serum (Gibco, Thermo Fisher Scientific, Inc., Waltham, MA, USA). Trif and Myd88 deficient RAW264.7 cells were generated using a clustered regularly interspaced short palindromic repeats (CRISPR)/CRISPR‐associated protein 9 system. Single guide RNAs were cloned into a pcDNA3.1 eukaryotic expression vector and transfected with CRISPR‐associated protein 9 plasmid into RAW264.7 cells, followed by selection with blasticidin and puromycin and then single‐cell cloning.

### *In vitro Listeria monocytogenes* infection of macrophages

Cells were infected with *L. monocytogenes* (Dr H. Shen from University of Pennsylvania School of Medicine, Philadelphia, PA, USA) at a multiplicity of infection of 10 for 30 min. To determine the number of bacteria entering the cells, extracellular bacteria were killed by treatment with 100 µg·mL^−1^ gentamicin (Sigma‐Aldrich, St Louis, MO, USA) for an additional 30 min. Then, infected cells were washed with PBS three times and lysed with 0.05% Triton X‐100 (Sigma‐Aldrich). Serial dilutions were seeded on brain‐heart infusion agar plates and colony‐formed units were counted after 36 h.

### RNA interference

Small interfering RNA (siRNA) (5 nmol) (Dharmacon; Thermo Fisher Scientific, Inc.) was transfected into cells using Lipofectamine RNAiMAX (Thermo Fisher Scientific, Inc) in accordance with the manufacturer’s instructions. After 48 h post‐transfection, cells were treated with LPS for the indicated time.

### Plasmid constructs

Recombinant vectors encoding mouse RNF10 were constructed by PCR‐based amplification from cDNA of mouse macrophages and then were subcloned into the pcDNA3.1 eukaryotic expression vector (Invitrogen; Thermo Fisher Scientific, Inc.). RNF10 promoter (~2000 bp upstream of the ATG initiation codon of the mouse Rnf10 gene) was constructed by PCR‐based amplification from genomic DNA of mouse macrophages followed by subcloning into the pGL‐basic vector (Invitrogen). All constructs were confirmed by DNA sequencing.

### Quantitative reverse transcriptase‐PCR

Total RNA was extracted with TRIzol reagent (Invitrogen) in accordance with the manufacturer’s instructions. For reverse transcriptase‐PCR, the cDNA was synthesized by a Reverse Transcription System (TransGen Biotech, Beijing, China). Reverse transcription products of different samples were amplified by LightCycler System (Roche, Basel, Switzerland) using SYBR Green PCR Master Mix (Vazyme Biotech Co., Nanjing, China) in accordance with the manufacturer’s instructions and the data were normalized by the level of β‐actin expression in each individual sample. The 2^–ΔΔct^ method was used to calculate relative expression changes. The gene‐specific primers were:

Rnf10 forward, 5'‐CCGGCGAGTCTAAACCCAAG‐3',

Rnf10 reverse, 5'‐CGACGGGACTGGTTGCTAAAA‐3',

Ifna4 forward, 5'‐TGATGAGCTACTACTGGTCAGC‐3',

Ifna4 reverse, 5'‐GATCTCTTAGCACAAGGATGGC‐3',

Ifnb1 forward, 5'‐ CAGCTCCAAGAAAGGACGAAC‐3',

Ifnb1 reverse, 5'‐ GGCAGTGTAACTCTTCTGCAT‐3',

Il1b forward, 5'‐GCAACTGTTCCTGAACTCAACT‐3',

Il1b reverse, 5'‐ATCTTTTGGGGTCCGTCAACT‐3',

Il6 forward, 5'‐TAGTCCTTCCTACCCCAATTTCC‐3',

Il6 reverse, 5'‐TTGGTCCTTAGCCACTCCTTC‐3',

Tnfa forward, 5'‐ GACGTGGAACTGGCAGAAGAG‐3',

Tnfa reverse, 5'‐ TTGGTGGTTTGTGAGTGTGAG‐3',

β‐actin forward, 5'‐AGTGTGACGTTGACATCCGT‐3',

β‐actin reverse, 5'‐GCAGCTCAGTAACAGTCCGC‐3'.

### Assay of luciferase reporter gene expression

Luciferase reporter plasmids for NF‐κB and interferon regulatory factor 3 (IRF3) have been described previously [22, 23]. HEK293 cells were transfected with a mixture of the appropriate luciferase reporter plasmid, pRL‐TK‐Renilla luciferase plasmid and the appropriate additional constructs. The total amount of plasmid DNA was made equal by the addition of an empty control vector. After 24 or 36 h, cells were left untreated or were treated with LPS (0111:B4). Luciferase activity was measured with a Dual‐Luciferase Reporter Assay System in accordance with the manufacturer's instructions (Promega, Madison, WI, USA). Relative luciferase activities were calculated as the ratio of firefly to Renilla luciferase light unit. Each luciferase activity value was the average of three independent experiments.

### Western blot analysis

Cells were lysed with cell lysis buffer (Cell Signaling Technology, Beverly, MA, USA) supplemented with protease inhibitors. Protein concentrations in the extracts were measured by a BCA assay (Pierce, Rockford, IL, USA). Immunoprecipitation analysis was performed as described previously [22]. Antibodies specific to p65 (#8242S), IRF3 (#4302S), Toll/IL‐1 receptor domain‐containing adaptor inducing IFN‐β (TRIF) (#4596), glyceraldehyde‐3 phosphate dehydrogenase (#5174), phosphorylated p65 (#3033S) and phosphorylated IRF3 (#4947S) were obtained from Cell Signaling Technology. Antibody anti‐RNF10 (ab104159) was obtained from Abcam (Cambridge, UK). Horseradish peroxidase‐coupled secondary antibodies were from Santa Cruz Biotechnology (Santa Cruz, CA, USA).

### Statistical analysis

Data were expressed as the group mean ± SD of one experiment representative of two to four identical experiments in which similar results were obtained. *P* < 0.05 (*t*‐test) was considered statistically significant.

## Results

### The expression of RNF10 is decreased in aged mouse macrophages

**Fig. 1:**
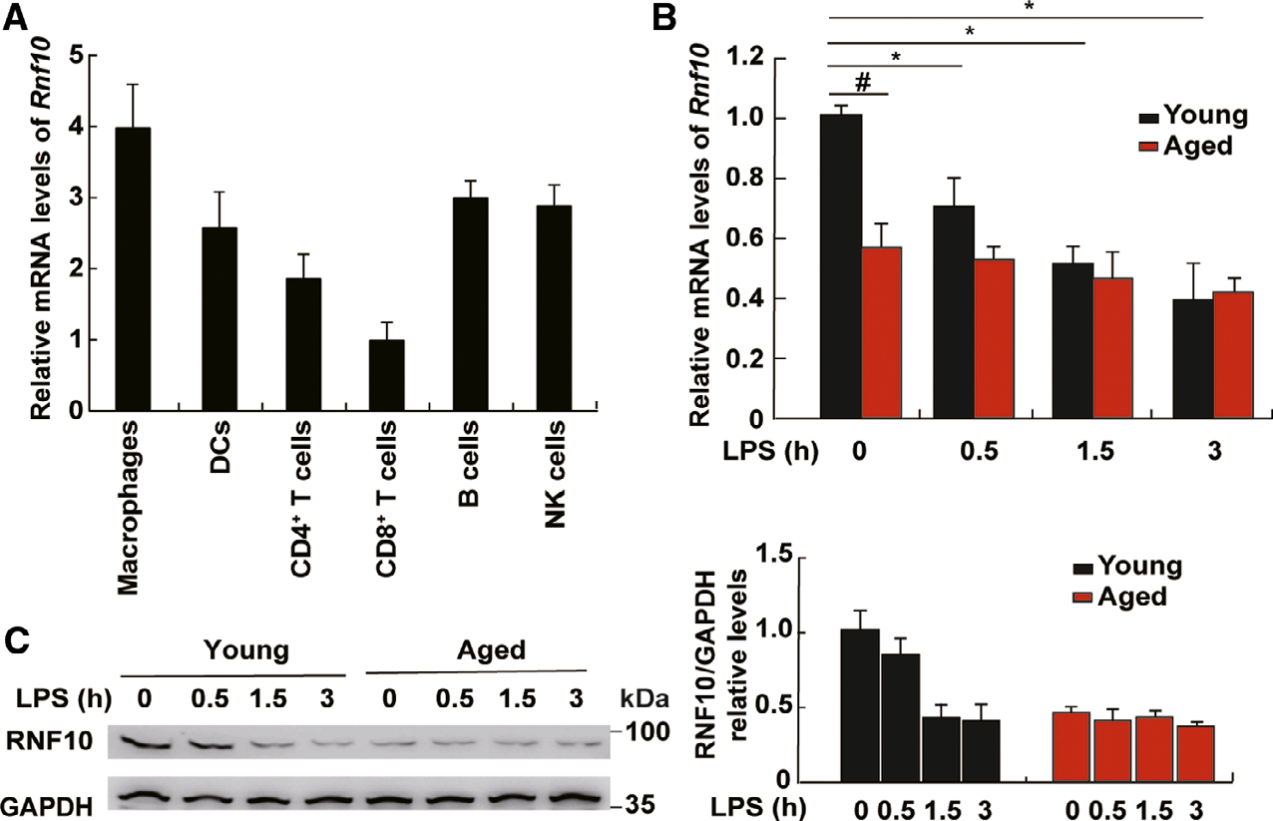
The expression of RNF10 in cells from young and aging mice. (A) Quantitative‐PCR analysis of RNF10 expression in murine immune cells [macrophages, DCs, CD4^+^ T cells, CD8^+^ T cells, B cells and natural killer (NK) cells]. (B) Quantitative‐PCR analysis of RNF10 expression in macrophages of aged mice and young mice stimulated with LPS. (C) Immunoblot analysis of RNF10 expression in macrophages of aged mice and young mice stimulated with LPS (left). The relative protein levels were determined using imagej (NIH, Bethesda, MD, USA) (right). Data are representative of independent experiments. Data are shown as the mean ± SD (*n* = 3). *P < 0.05 (Student’s *t*‐test).

We detected the expression of RNF10 in immune cells, including macrophages, DCs, CD4^+^ T cells, CD8^+^ T cells, B cells and natural killer cells. It was revealed that RNF10 was highly expressed in all immune cells, especially in macrophages (Fig. 1A). Then, we treated primary macrophages from aged or young mice with LPS for different times and observed the expression of RNF10. Under physiological conditions, the mRNA and protein levels of RNF10 were both higher in young mouse macrophages compared to aged mouse cells. After LPS treatment, RNF10 expression was markedly declined in young cells in a time‐dependent manner, whereas no obvious difference was observed in aged mouse cells (Fig. 1B,C). Next, we investigated whether reduced RNF10 expression is associated with age‐related inflammatory responses.

### RNF10 expression is under the regulation of TRIF

**Fig. 2:**
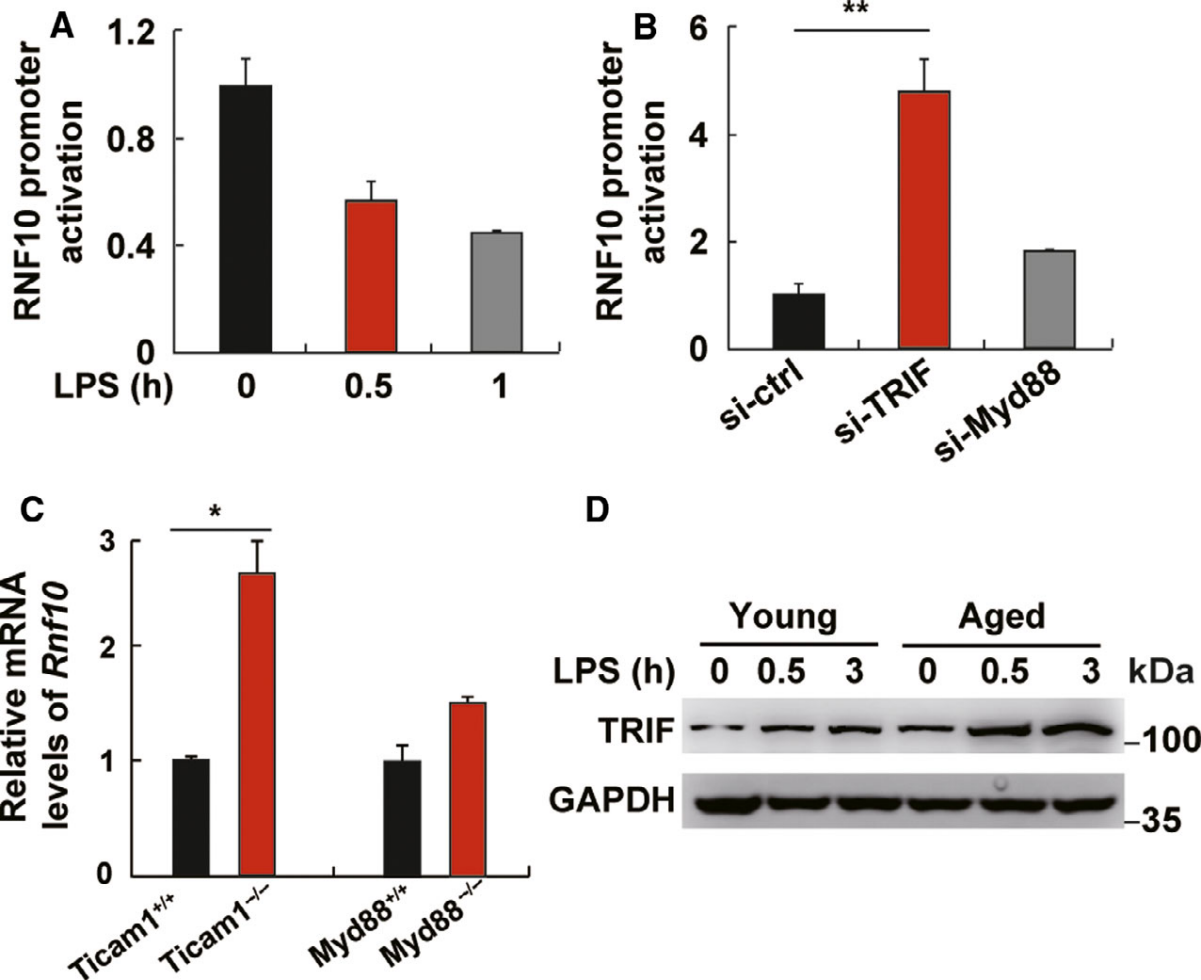
The promoter activity of RNF10 is regulated by TRIF. (A) RNF10 promoter activity in L929 cells treated with LPS for 0.5 and 1 h. (B) RNF10 promoter activity in L929 cells transfected with si‐ctrl, si‐TRIF or si‐Myd88 for 48 h before LPS stimulation for 1 h. (C) Quantitative‐PCR analysis of RNF10 expression in wild‐type, Ticaml^–/–^ and Myd88^−/−^ RAW264.7 cells. (D) Immunoblot analysis of TRIF in peritoneal macrophages from young and aged mice stimulated with LPS for the indicated time. Data are representative of independent experiments. Data are shown as the mean ± SD (*n* = 3). *P < 0.05, **P < 0.01 (Student’s *t*‐test).

To explore the underlying mechanism for the regulation of RNF10, we constructed the promoter of RNF10 and found that LPS mostly decreased the activity of the RNF10 promoter (Fig. 2A). Because LPS can activate both the TRIF‐dependent and Myd88‐dependent pathway, we detected which pathway was involved in the regulation of RNF10 expression. The results showed that the silencing of TRIF but not Myd88 markedly enhanced the activity of the RNF10 promoter (Fig. 2B). The finding was also observed in TRIF‐ and Myd88‐deficient RAW264.7 cells (Fig. 2C) because RNF10 demonstrated a higher expression in Trif‐ but not Myd88‐deficient cells, consistent with the finding in peripheral blood mononuclear cells (GEO profile: ID: 98365504). Taken together, these data confirm that RNF10 is under the regulation of the TRIF pathway but not the MyD88 pathway.

Additionally, we demonstrated that the expression of TRIF was higher in aged mouse macrophages compared to young cells without LPS stimulation. Furthermore, the expression of TRIF in aged mouse macrophages was much higher than in young cells upon LPS stimulation (Fig. 2D), which indicates that the expression of RNF10 may be inhibited by increased TRIF pathway activation in aged mouse macrophages and was consistently lower regardless of LPS treatment.

### Decreased RNF10 expression enhances the expression of proinflammatory cytokines

**Fig. 3:**
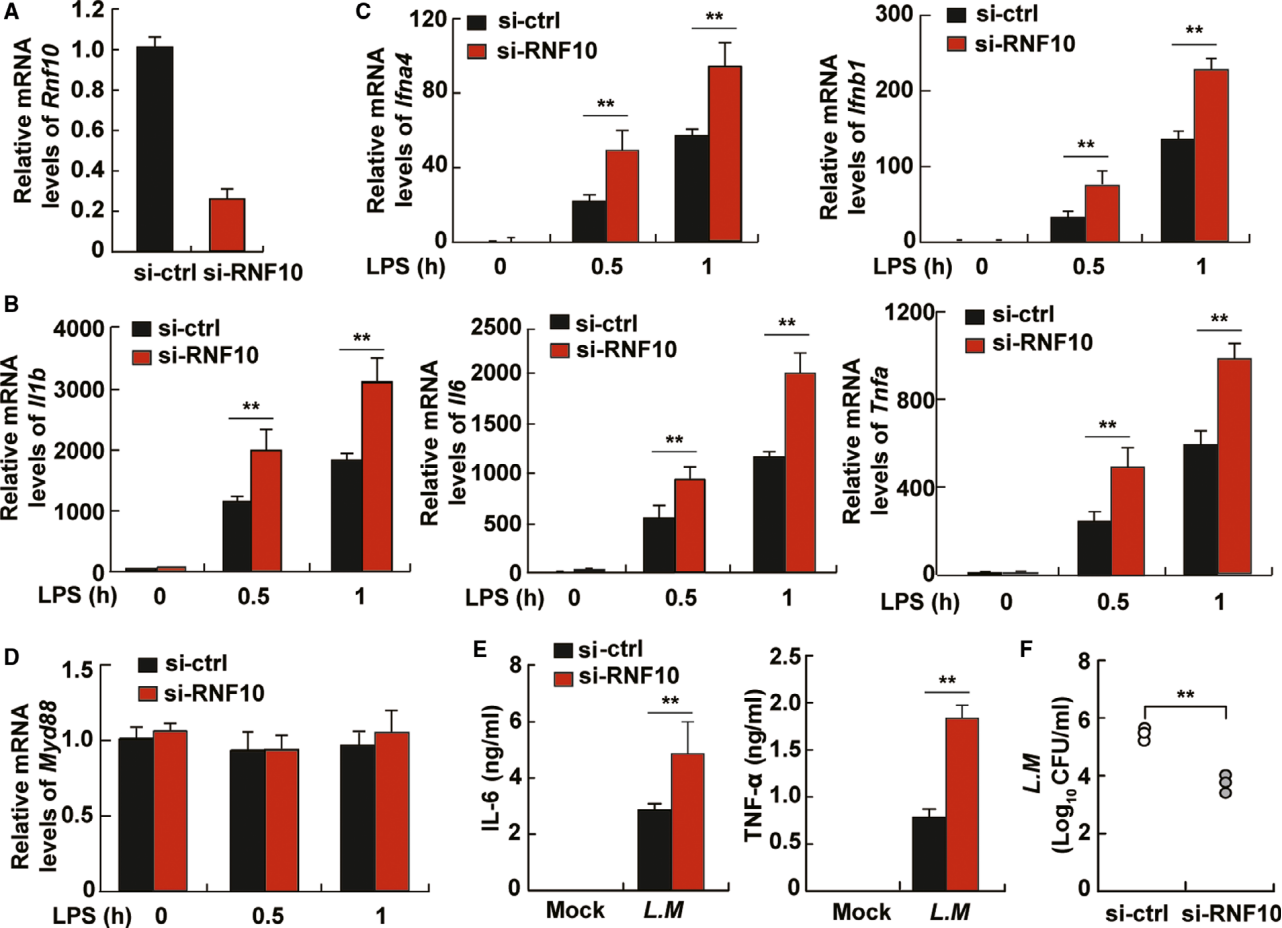
RNF10 inhibits the production of inflammatory cytokines in TLR‐triggered macrophages. (A) Quantitative‐PCR analysis of RNF10 mRNA expression in peritoneal macrophages transfected with control siRNA (si‐ctrl) or si‐RNF10 for 48 h. Quantitative‐PCR analysis of Il1b, Il6 and Tnfa expression (B), Ifna4 and Ifnb1 expression (C), and Myd88 expression (D) in peritoneal macrophages transfected with si‐ctrl or si‐RNF10 for 48 h and LPS stimulation for the indicated hours. (E) ELISA of IL‐6 and TNF‐α in supernatants of peritoneal macrophages transfected with si‐ctrl or si‐RNF10 for 48 h and *Listeria monocytogenes* infection at a multiplicity of infection of 10 for 6 h. (F) Colony‐formed units (CFUs) obtained from cells in (E). Data are representative of independent experiments. Data are shown as mean ± SD (*n* = 3). *P < 0.05, **P < 0.01 (Student’s *t*‐test).

To further investigate the role of RNF10 in the innate immune response, we adopted siRNA for knockdown of RNF10 in macrophages (Fig. 3A). The results showed that LPS‐induced IL‐1β, TNFα and IL‐6 (Fig. 3B), which were under the control of NF‐κB, as well as interferon (Ifn) a4 and Ifnb1 (Fig. 3C), were all increased when RNF10 was silenced using siRNA targeting RNF10 (si‐RNF10). However, the expression of Myd88, which was not affected in LPS‐treated macrophages, was not affected by RNF10 (Fig. 3D), suggesting that RNF10 may specifically contribute to the LPS‐triggered signaling pathway.

To determine the effect of RNF10 on pathogen clearance, we assessed the possible role of RNF10 in innate immune responses during bacterial pathogenesis. We performed an infection with Gram‐positive bacteria *L. monocytogenes* at a multiplicity of infection of 10 in macrophages and found that the silencing of RNF10 showed a higher production of IL‐6 and TNF‐α after infection for 6 h (Fig. 3E). Consistent with the results, bacterial proliferation was significantly lower in cells with si‐RNF10 as measured with colony‐formed units corresponding to intracellular live bacteria (Fig. 3F), suggesting that si‐RNF10 inhibits the proliferation of bacteria in macrophages. Taken together, our findings indicate that RNF10 has a negative role in the production of proinflammatory cytokines and the abnormal expression of RNF10 in macrophages from aged mice may be associated with delayed pathogen clearance.

### Decreased RNF10 expression promotes the NF‐κB and IRF3 signaling pathways

**Fig. 4:**
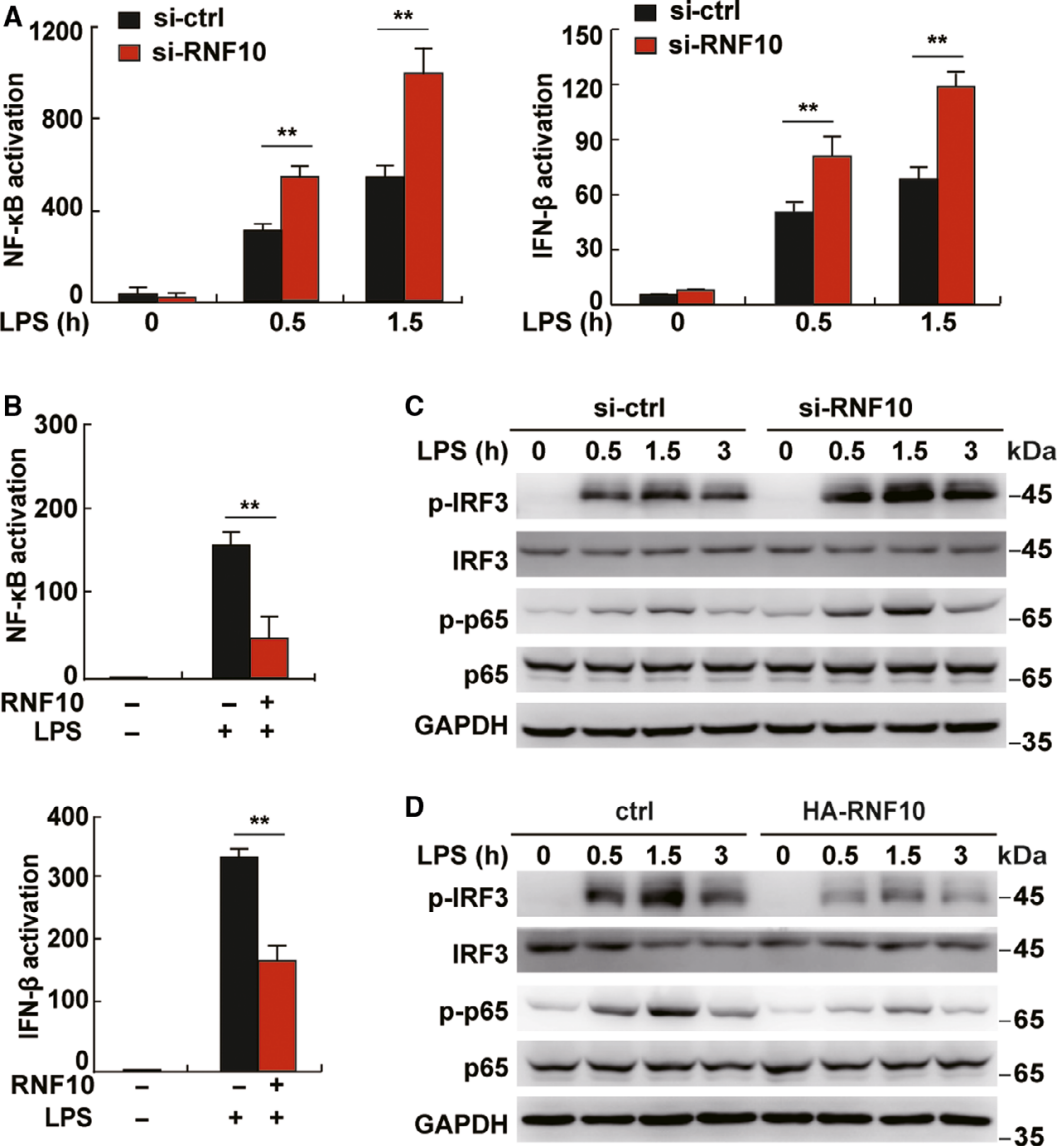
RNF10 inhibits the NF‐κB and IRF3 signaling pathways in LPS‐triggered macrophages. (A) NF‐κB and IFN‐β promoter activity in L929 cells transfected with si‐ctrl or si‐RNF10 for 48 h before LPS treatment for the indicated time. (B) NF‐κB and IFN‐β promoter activity in L929 cells transfected with overexpression of RNF10 vector for 48 h before LPS treatment for 1 h. (C) Immunoblot analysis of phosphorylated (p)‐IRF3 and p‐65 in macrophages transfected with si‐ctrl or si‐RNF10 for 48 h before LPS treatment for the indicated time. (D) Immunoblot analysis of p‐IRF3 and p‐65 in RAW264.7 cells transfected with RNF10 expression vector for 24 h before LPS treatment for 1 h. Data are representative of independent experiments. Data are shown as the mean ± SD (*n* = 3). *P < 0.05, **P < 0.01 (Student’s *t*‐test).

To further demonstrate the role of RNF10 in innate immune response, we investigated whether RNF10 could affect the phosphorylation of p65 and IRF3, which are usually regarded as indicators of NF‐κB and IFN‐β activity respectively. The results obtained demonstrated that knockdown of RNF10 could enhance NF‐κB activity and IFN‐β promoter activity upon LPS treatment (Fig. 4A). Correspondingly, overexpression of RNF10 decreased both activities (Fig. 4B). Furthermore, knockdown of RNF10 increased the level of phosphorylated p65 and phosphorylated interferon regulatory factor 3 in cells upon LPS treatment (Fig. 4C). Although overexpression of RNF10 significantly decreased both levels in cells upon LPS treatment (Fig. 4D), these results suggested that RNF10 negatively regulates the signal pathway of NF‐κB and IRF3 in response to LPS and also that aberrant RNF10 expression in aged mouse macrophages may be involved in age‐related defects in the inflammatory response.

## Discussion

The age‐related decline of the immune system has been generally associated with an increased susceptibility to infectious pathogens in old adults. However, the understanding of the mechanism governing the onset of immunosenescence remains incomplete. Here, we found that RNF10, a negative feedback regulator of Toll‐like receptor (TLR)‐induced signaling pathways, could be suppressed by increased TLR‐TRIF pathway activation and maintained at a lower expression level in macrophages of aged mice compared to young cells. Furthermore, the reduced expression of RNF10 increased the LPS‐triggered signaling pathways of NF‐κB and IRF3, thus enhancing proinflammatory cytokines and IFN‐I. Our results shed light on the regulatory mechanism of dysregulated expression of RNF10 in immunosenescence and unearth the potential clinical implications in multiple age‐related inflammatory diseases.

RNF10, a RING finger domain‐containing E3 ubiquitin ligase, was previously reported to suppress inflammatory responses, hyperproliferation [17, 18], regulation of apoptosis [17, 21, 24], cell differentiation [19], and neuronal development and morphology [25, 26]. In particular, RNF10 contains three putative nuclear localization signals [16] and acted as a synaptonuclear protein messenger located in the nucleus for modulation of synaptic transmission and synaptic plasticity [20, 26], or regulated gene transcription and myelin formation [25, 27]. However, other studies have reported that RNF10 in the cytoplasm interacted with other proteins and enhanced protein activation [28, 29]. Therefore, we speculated that the localization of RNF10 may depend on cell type, as has been shown for the control of nuclear localization of PBX1 in developing limbs [30]. Furthermore, the ectopic expression of RNF10 may lead to its accumulation in proteasomes localized in the cytoplasm. Considering that E3 ligase RNF10 inhibited the LPS‐triggered downstream phosphorylation of p65 and IRF3 in our study, we deduced that RNF10 may affect the ubiquitination of key proteins in the TLR4‐mediated downstream pathway if RNF10 is localized in the cytoplasm, or RNF10 may mainly affect the ubiquitination of dephosphorylase responsible for both p65 and IRF3, or the ubiquitinate proteins involved in cell metabolism or epigenetics if RNF10 is localized in the nucleus, Next, we aimed to determine the localization and the regulation mechanism of RNF10 in mouse macrophages.

Immunosenescence is characterized by persistent low‐grade chronic inflammation (inflammaging) and a decreased ability of the immune system to respond to foreign antigens in the elderly [31]. Many studies have demonstrated reduced TLR‐induced cytokine secretion in macrophages with aging [32], which may explain why the elderly have an increased susceptibility to bacterial, mycotic and viral infections. A number of negative signaling regulators, such as IRAK‐M, SOCS‐1, A20 and Tollip, have been shown to play an important role in TLR signal transduction [33]. It will be interesting to investigate whether aging impacts the expression, phosphorylation and function of these regulators, which could be a result of decreased mRNA stability, chromatin modification of the TLR genes or the dysfunction of transcription factors required for expression. Here, we demonstrated the negative role of RNF10 in the TLR‐induced production of proinflammatory cytokine and IFN‐I by activating transcription factors including NF‐κB and IRF3. In addition, abnormal TRIF expression was demonstrated to be associated with aberrant RNF10 expression in aged macrophages, indicating that RNF10 may act as a TRIF‐dependent inflammatory mediator. Our findings indicate that the aberrant expression of RNF10 was associated with age‐associated dysregulation of immune function in macrophages. However, it remains to be determined whether the restoration of RNF10 expression restores the antibacterial and wound healing functions of macrophages in aged mice. A more detailed understanding of the impact of RNF10 expression could enable us to develop strategies to overcome age‐related defects and provide improved disease control and prevention for the elderly.

In summary, our findings have demonstrated that dysregulated expression of RNF10 contributes to the age‐related dysfunction of macrophages, such that RNF10 might provide new insights into the mechanism of age‐related degenerative pathology. Our results reveal the underlying mechanism of immunosenescence, define a new inflammatory mediator under the TRIF pathway and lay the foundation for further research into clinical diseases associated with inflammatory aging.

## Conflict of interest

The authors declare no conflict of interest.

## Author contributions

YY designed and supervised the research. XC, LL and YZ performed the experiments. XC and YY analyzed data and wrote the paper. All authors reviewed and revised the manuscript, and approved the final version submitted for publication. All authors agree to be accountable for all aspects of the work.

## Data Availability

The data are available from the corresponding author upon reasonable request.
